# Breast Reconstruction After Cancer: Historical Development, Modern Techniques, and Psychological Impact

**DOI:** 10.3390/healthcare14091140

**Published:** 2026-04-24

**Authors:** Maks Tušak, Aleš Porčnik, Ivan Kneževič, Jasmina Markovič-Božič, Matej Tušak, Andrej Lapoša

**Affiliations:** 1 Surgical Clinic, University Medical Centre Ljubljana, 1000 Ljubljana, Slovenia; 2 Department of Plastic, Reconstructive, Aesthetic Surgery and Burns, University Medical Centre Ljubljana, 1000 Ljubljana, Slovenia; ales.porcnik@kclj.si (A.P.); andrej.laposa@kclj.si (A.L.); 3 Department of Surgery, Faculty of Medicine, University of Ljubljana, 1000 Ljubljana, Slovenia; 4 Transplantation Centre, University Medical Centre Ljubljana, 1000 Ljubljana, Slovenia; ivan.knezevic@kclj.si; 5 Department of Anaesthesiology and Surgical Intensive Therapy, University Medical Centre Ljubljana, 1000 Ljubljana, Slovenia; jasmina.markovic1@kclj.si; 6 Department of Anaesthesiology and Reanimation, Faculty of Medicine, University of Ljubljana, 1000 Ljubljana, Slovenia; 7 Science and Research Centre Koper, 6000 Koper, Slovenia; tusakm@gmail.com

**Keywords:** breast reconstruction, mastectomy, free flap, reconstruction methods, DIEP flap, autologous breast reconstruction, reconstruction with breast implants

## Abstract

Breast reconstruction represents an integral component of contemporary breast cancer management, with substantial impact on patients’ psychological well-being, body image, and overall quality of life. Given the profound symbolic and personal significance of the breast, mastectomy—whether total or partial—extends beyond oncologic resection and may result in considerable aesthetic, functional, and psychosocial consequences. For this reason, reconstructive planning should be incorporated into the initial multidisciplinary treatment strategy while ensuring that oncologic safety and adjuvant therapies are never compromised. Breast reconstruction may be achieved using autologous tissue, implant-based techniques, or a combination of both approaches. Each method carries specific advantages, limitations, and potential complications and must be tailored to the individual patient’s oncologic status, anatomy, and expectations. This article provides a historical overview of the evolution of breast cancer treatment and reconstructive techniques. It further examines the principles, benefits, and challenges associated with different reconstructive modalities, highlighting key considerations in clinical decision-making and long-term outcomes.

## 1. Introduction

The breast is an important symbol of femininity, motherhood, and fertility. It is, therefore, completely understandable that losing this symbol because of breast cancer—whether through mastectomy or tumorectomy—can be a significant aesthetic and emotional burden for a woman. A considerable proportion of patients (15–30%) continue to struggle with their (physical) self-image for some time after completing treatment, they may also experience fear of recurrence, which represents a risk factor for developing mental health disorders such as anxiety and depression [1,2]. Poor self-image can lead to sexual difficulties, problems within a partnership, and an overall reduced quality of life [1,2]. In addition to scarring, changes caused by systemic (non-surgical) treatments—such as weight gain, lymphedema, hot flashes, vaginal dryness, fatigue, and various cognitive problems—can also contribute to a negative body image [3]. Altered self-perception may even manifest as preoccupation with one’s appearance or unrealistic expectations regarding surgical procedures [4].

Breast reconstruction is an important reconstructive option that can significantly improve a patient’s psychological well-being and overall quality of life. It enhances a woman’s self-image and can, in turn, have a positive effect on her relationship with her partner. It is essential to consider breast reconstruction as part of the initial treatment plan; however, the reconstruction itself must never compromise oncologic therapy. The decision to proceed with reconstruction must always be confirmed by a multidisciplinary medical team.

In modern breast cancer centers, reconstruction is an integral part of treatment. Breast reconstruction may be performed using autologous techniques, where the patient’s own tissue is used, with implant-based techniques, or with a combination of both.

## 2. Methods

This narrative review was undertaken to provide a structured overview of the historical development, current reconstructive techniques, and psychological aspects of breast reconstruction after breast cancer surgery. A targeted literature search was performed using PubMed and Web of Science to identify relevant English-language publications. The search strategy included terms such as breast reconstruction, implant-based reconstruction, autologous reconstruction, DIEP flap, TRAM flap, immediate reconstruction, delayed reconstruction, and psychological outcomes. Articles were selected on the basis of their relevance to the scope of the review, with particular emphasis on reconstructive techniques, long-term outcomes, and patient-reported psychological and functional outcomes. In addition to the available literature, the review also reflects the authors’ clinical experience at the University Medical Centre Ljubljana, Ljubljana, Slovenia including institutional perspectives on commonly used reconstructive strategies and approach selection in specific clinical contexts.

## 3. Treatment of Breast Cancer—Past and Present

References from very ancient civilizations show that breast cancer has been known for a long time and that in those times it was considered incurable. Breast tumors were described as early as 3000 BC in Egypt. On the Egyptian papyrus known as the Edwin Smith Papyrus (named after its collector), which is the oldest known surgical treatise, one can find descriptions of breast cancer cases and even treatments, such as cauterization with a burning rod; yet ultimately it was concluded that the disease was incurable.

Later, the ancient Greeks and Romans described breast cancer (the Greeks referred to it as carcinoma, due to its crab-like appearance), and they also proposed—and carried out—various surgical techniques to remove the breast. Aside from amputating the breast tissue, they sometimes used a heated rod to burn tissue and thus stop bleeding. During the Middle Ages, advances in breast surgery stagnated. In the 16th and 17th centuries, however, surgery re-emerged—this time in the form of brutal “guillotine” mastectomies. The 17th-century surgeon Johann Shultes is considered a pioneer of the guillotine mastectomy; he even described specialized instruments for his procedure. From today’s perspective, all these methods—including those from the 16th and 17th centuries—were extremely harsh. Lacking proper antiseptics and anesthesia, the success of surgery depended on speed rather than precision. A “good surgeon” at that time was valued for how quickly he could operate—not for fine technique.

Until the 19th century, mastectomy remained a dreadful procedure, associated with agony, prolonged recovery, and a profound impact on the physical and psychological well-being of the patient—a suffering vividly described by the famous writer Fanny Burney, who underwent such a mastectomy herself [5].

With the introduction of antisepsis toward the end of the 19th century, survival after surgical treatment of breast cancer began to improve. A major milestone in breast cancer treatment was set by William Stewart Halsted, the American surgeon who first described a radical procedure removing the entire breast tissue, both pectoral muscles, and the axillary lymph nodes—a method that became the gold standard for the next hundred years [6]. Unfortunately, Halsted’s view also hindered the development of reconstructive techniques: he believed that reducing the amount of excised tissue for the sake of reconstruction could compromise complete removal of cancerous tissue or increase the risk of recurrence.

Since the 1980s, surgical treatment of breast cancer has evolved significantly. Radical mastectomy has been modified so that both the major and minor pectoral muscles are preserved. At the same time, for smaller tumors, breast-conserving surgeries (such as quadrantectomy or tumorectomy) became common. In these procedures, only the portion of the gland containing the tumor—together with a safety margin of glandular tissue, surrounding fat, and skin—is removed. In 1991, the technique of skin-sparing mastectomy was described.

More recently, for selected indications, surgeons increasingly perform nipple sparing mastectomy, where only the glandular and fat tissue of the breast is removed while preserving the overlying skin, the areola, and the nipple. Previously, for select cases, only skin-sparing mastectomy (preserving skin but not nipple/areola) was done. Today’s breast cancer treatment is multimodal, involving surgery, systemic therapy, and radiotherapy.

An essential part of modern management is also breast reconstruction. If a multidisciplinary board approves reconstruction and the patient desires it, she may undergo the procedure. In Slovenia, the foundations of microsurgery were laid by Marko Godina, who described numerous techniques now recognized worldwide in plastic and reconstructive surgery. His work in the field of microsurgical reconstruction is continued at the Plastic, Reconstructive and Aesthetic Surgery Department of University Medical Centre Ljubljana, including reconstructive breast surgery. Today, breast cancer treatment is truly multidisciplinary: oncologists collaborate with plastic and reconstructive surgeons to ensure the best possible oncologic and reconstructive outcomes.

## 4. History of Breast Reconstruction

Surgical treatment of breast cancer left women physically disfigured, with lasting consequences for psychological well-being and sexual health. Axillary lymph-node removal often resulted in impaired lymphatic drainage of the upper limb, while chronic pain and reduced shoulder mobility were additional problems faced by patients after mastectomy. It is therefore not surprising that early ideas about surgical breast reconstruction began to emerge.

The first documented breast reconstruction dates back to 1895, when Czerny [7] transplanted a lipoma from the patient’s lower back into the defect created after mastectomy to correct breast asymmetry. This initial reconstruction was thus autologous, but ultimately unsuccessful because the lipomatous tissue became necrotic due to lack of vascular supply. Ten years later, Ombredanne [8] used the pectoralis muscle for breast reconstruction, molding the muscle mass to mimic the shape of a breast. In 1906, Tanzini [9] introduced the first myocutaneous flap for breast reconstruction—the latissimus dorsi flap with an overlying skin island—primarily to achieve reliable wound closure after mastectomy. Modified versions of this technique are still used today.

The first free flap for breast reconstruction was performed by Holmström [10] in 1979, using a free transverse rectus abdominis muscle (TRAM) flap (then referred to as a free abdominoplasty flap). This represented the first microsurgical procedure in breast reconstruction and marked a major turning point, even though microsurgery was far from routine at that time. Autologous breast reconstruction truly flourished after Hartrampf published his experience in 1982 [11] with the pedicled TRAM flap. Tissue from the lower abdomen was transferred to the mastectomy site without dividing the epigastric artery and vein. Since then, abdominal tissue has become the most common donor site for autologous breast reconstruction. The pedicled TRAM flap served as the driving force for further development. As microsurgery advanced, the pedicled TRAM evolved into its free-flap form (although Holmström had already demonstrated it years earlier). Two further refinements followed: the muscle-sparing TRAM (ms-TRAM) flap, which sacrifices only a small portion of the muscle, and the deep inferior epigastric perforator (DIEP) flap [12,13], in which the muscle is preserved entirely. Both techniques are performed in our setting, with the DIEP flap—requiring no harvest of abdominal wall muscle—now considered the gold standard in autologous breast reconstruction.

The DIEP flap was first described by the Japanese microsurgeon Koshima in 1989 [12], but it was Allen and Treece [13] who first used it for breast reconstruction in 1994. Their technique remains the basis of current practice: perforating vessels are identified above the rectus sheath and traced through the anterior sheath and muscle fibers to their origin from the deep inferior epigastric artery (DIEA).

In 2011, Ahčan [14] performed the first breast reconstruction using a 3D model to guide shaping of the abdominal flap in order to match the contralateral breast.

In 1961, Cronin and Gerow [15] developed the first silicone gel-filled breast implant. The first implantation was performed in 1962, introducing a prosthesis composed of a silicone elastomer shell filled with viscous silicone gel. This innovation provided a more natural consistency and contour compared with earlier reconstructive attempts, which had relied on autologous tissue transfers, local flaps, or synthetic materials (e.g., paraffin wax, polyurethane foam, polyvinyl alcohol sponge) associated with high complication rates. The advent of silicone implants significantly expanded reconstructive possibilities and laid the foundation for the development of implant-based breast reconstruction as a standard technique.

A decade later, Radovan [16] introduced the concept of the tissue expander, originally designed for patients with extensive soft-tissue loss. The device consists of a temporary inflatable implant equipped with a filling port, allowing for gradual saline expansion over time. By progressively stretching the overlying skin and soft tissues through controlled mechanical expansion, a sufficient tissue envelope can be generated to accommodate a definitive implant.

## 5. Selection of the Reconstruction Method

When selecting the method of breast reconstruction, the patient’s wishes must be carefully considered. Together with the patient, the most appropriate reconstructive option is chosen. It is essential to determine the optimal timing of reconstruction and to incorporate reconstructive planning into the initial treatment strategy. Close collaboration between the oncologist and the reconstructive surgeon is crucial.

The choice of reconstruction must take into account the stage of disease and the planned oncological treatment (e.g., the need for postoperative radiotherapy), the patient’s general health status and comorbidities (such as diabetes mellitus), lifestyle factors (e.g., smoking), the size and degree of ptosis of the contralateral breast, as well as the patient’s body characteristics (body mass index and the availability of tissue for autologous reconstruction). In addition, the condition of the tissues in the mastectomy area must be assessed, including the presence of scars, tissue perfusion, radiation-related changes, and the thickness of the subcutaneous fat layer.

The following considerations serve as the main guiding principles when choosing between implant-based and autologous breast reconstruction. Implant-based reconstruction is generally preferred in patients seeking a less invasive procedure, shorter operative time, and faster recovery, particularly when adequate soft-tissue coverage is present and the anticipated need for postoperative radiotherapy is low. In contrast, autologous reconstruction is often favored in patients who are expected to require radiotherapy, in those with sufficient donor-site tissue, and in patients desiring a more natural and durable long-term result.

At the University Medical Centre Ljubljana, reconstructive options and potential complications are discussed by the patient, the oncologic surgeon, and the reconstructive surgeon within a multidisciplinary onco-reconstructive board, where a consensus is reached regarding the most suitable reconstructive method for the patient. The final decision regarding reconstruction is always made by the patient, based on the information provided during the onco-reconstructive consultation. Patients are also offered the opportunity to speak with trained volunteers who have successfully undergone breast cancer treatment and breast reconstruction.

## 6. Timing of Breast Reconstruction

Breast reconstruction can be classified according to the timing of the procedure into immediate and delayed reconstruction. Immediate (primary) reconstruction is performed directly following tumor resection as a continuation of the oncologic operation, whereas delayed (secondary) reconstruction is undertaken after completion of oncologic treatment as a separate surgical procedure.

**Figure 1:**
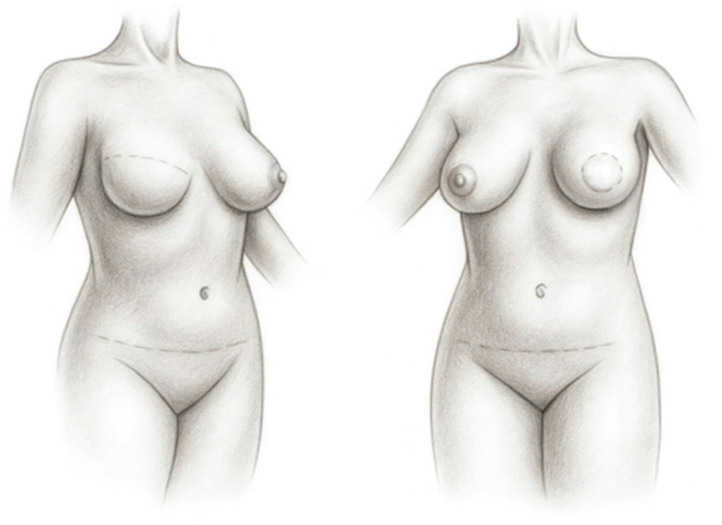
Outcome and scarring after delayed (**left**) and immediate (**right**) autologous breast reconstruction with a free flap (DIEP flap).

Immediate reconstruction is carried out during the same operative session as total or partial mastectomy. This approach is generally considered the preferred option, as it reduces the psychological burden associated with the loss of the breast and eliminates the period between mastectomy and reconstruction. In addition, aesthetic outcomes are often superior, with fewer scars or with scars that can be placed more discreetly (Figure 1). In many patients undergoing immediate reconstruction, the breast skin envelope and inframammary fold can be preserved (and in some cases even the nipple–areola complex), which significantly influences the final reconstructive outcome. However, immediate reconstruction requires careful coordination of a multidisciplinary team and may be technically more demanding; therefore, it is typically performed in larger, well-organized specialized centers.

Delayed reconstruction may be performed months or even years after mastectomy, most commonly after completion of adjuvant oncologic therapy (radiotherapy), usually within three to six months. An advantage of the delayed approach is that it allows more time for thorough preoperative planning and for selection of the most appropriate reconstructive method. It also provides the patient with sufficient time to understand the benefits and limitations of the available options, enabling more active participation in the decision-making process.

In recent years, so-called delayed–immediate reconstruction has also been used as an additional reconstructive strategy. The choice of adjuvant oncologic treatment, including systemic therapy and radiotherapy, is largely determined by the final histopathologic evaluation of the tissue removed at mastectomy. To preserve the native breast skin envelope and contour, a saline-filled tissue expander is placed immediately after mastectomy as a temporary measure. If final pathology indicates that radiotherapy is not required, definitive reconstruction may proceed in a second stage soon thereafter. Conversely, if radiotherapy is indicated, reconstruction is deferred until completion of irradiation and is then preferably performed using autologous tissue, when feasible.

## 7. Methods of Breast Reconstruction

According to the reconstructive technique, breast reconstruction may be performed using autologous tissue, in which the patient’s own tissue is transferred, or with alloplastic materials, including the use of a tissue expander followed by placement of a permanent anatomically shaped silicone implant. Combined approaches are less commonly used but may be considered in selected patients to optimize reconstructive outcomes.

## 8. Autologous Breast Reconstruction

Autologous breast reconstruction involves restoration of the breast using the patient’s own tissues. A segment of skin and subcutaneous fat, and in selected cases a portion of muscle, is harvested from another region of the body, either transferred as a pedicled flap or completely detached and revascularized using microsurgical techniques, and subsequently inset at the site of the mastectomy defect. This allows formation of a new breast mound that closely approximates the native breast in both shape and volume.

The abdomen represents the most common donor site, although tissue may also be harvested from the thighs, back and even intra-abdominally (omentum flap). A breast reconstructed with autologous tissue behaves physiologically like normal soft tissue: it maintains body temperature and changes in volume with weight fluctuations. In most women over the age of 40, the lower abdomen provides sufficient tissue with characteristics similar to those of the breast, which is why flaps from this region are most frequently used. Harvesting tissue from the lower abdomen additionally results in contouring of the abdominal wall, effectively producing an effect comparable to an abdominoplasty, which may represent an added benefit for some patients.

Autologous reconstruction generally provides the most natural breast shape, consistency, and ptosis, and achieves good symmetry with the contralateral breast. The results are durable over time, as the transferred tissue ages and responds to weight changes in a manner similar to native breast tissue. Consequently, secondary corrective procedures are often unnecessary. In patients with adequate abdominal tissue requiring bilateral breast reconstruction, bilateral autologous reconstruction can be performed, although the reconstructed breasts may be smaller in volume. When abdominal tissue is insufficient, particularly in patients with smaller breasts, tissue from the gluteal region or the inner thigh may be considered as an alternative donor site.

## 9. Autologous Breast Reconstruction with a Free Flap (Microsurgical Reconstruction)

Autologous breast reconstruction using a free flap represents the most advanced technique in autologous breast restoration. Although it requires specialized microsurgical expertise, its numerous advantages have established it as the gold standard for reconstruction with autologous tissue. In this approach, tissue together with its vascular pedicle supplying arterial inflow and venous outflow is completely detached from the donor site and transferred to the recipient site. Using an operating microscope and microsutures, microvascular anastomoses are then performed between the flap vessels and recipient vessels.

Compared with pedicled flaps, free flaps offer several advantages, the most important being superior perfusion of the transferred tissue [17]. Additional benefits include reduced weakening of the abdominal wall, the possibility of harvesting a larger volume of tissue, and greater flexibility in shaping the breast mound, resulting in improved symmetry and aesthetic outcomes.

As early as 1983, Scheflan and Dinner [18] reported poor perfusion in zones III and IV of the pedicled TRAM flap. Similar observations were later described by Smith and colleagues [19] and Harashina and colleagues [20]. Compromised vascularity may lead to skin and fat necrosis, as the principal blood supply to the periumbilical and infraumbilical regions is derived from the inferior epigastric system [21]. Furthermore, when a pedicled flap is transposed to the chest, rotation of the muscle through a narrow subcutaneous tunnel may compress small vascular connections between adjacent perfusion territories, thereby increasing the risk of complications [21].

Microsurgical breast reconstruction is the most technically demanding form of breast reconstruction. The procedure typically lasts three to five hours and requires surgeons skilled in microsurgical techniques. It provides durable results and yields the most natural breast shape and tissue characteristics. A prerequisite for free-flap reconstruction is an adequate volume of donor tissue. Not infrequently, particularly in older patients, the reconstructed breast is smaller than the contralateral side, necessitating a secondary symmetrization procedure such as reduction or mastopexy of the opposite breast.

The abdomen is the most commonly used donor site because it provides abundant adipose tissue with consistency similar to that of the breast.

The most frequently used free flaps for autologous breast reconstruction include the DIEP flap, superficial inferior epigastric artery (SIEA) flap, free TRAM flap, superior gluteal artery (SGA) flap, inferior gluteal artery (IGA) flap, and the deep circumflex iliac artery (DCIA) flap or “Rubens” flap. The transverse upper gracilis (TUG) flap, based on the vascular pedicle of the gracilis muscle, and the PAP flap, based on perforators of the profunda femoris artery, may also be used. The DIEP flap is widely regarded as the gold standard of autologous reconstruction and is the technique most commonly employed in our practice.

**Figure 2:**
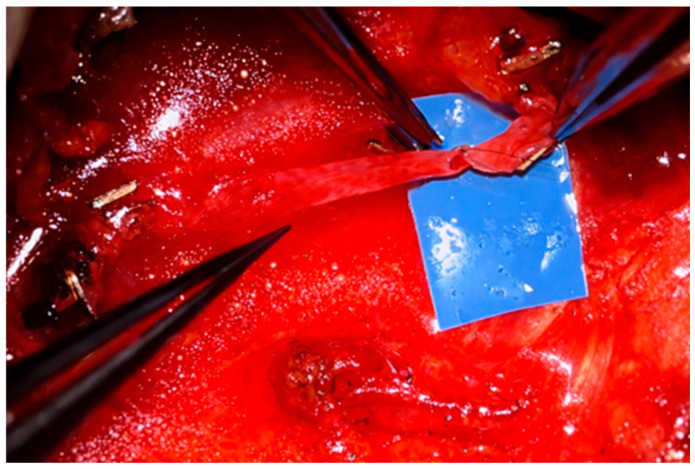
Suturing of a microvascular anastomosis between the recipient vessel and the flap vessel.

In immediate autologous reconstruction (performed during the same operation as mastectomy) the oncologic surgeon removes the breast gland, nipple–areola complex, and most often preserves the skin envelope (skin-sparing mastectomy). The reconstructive surgeon then transfers a free flap from the donor site—most commonly the abdomen—into the resulting defect and performs microvascular anastomoses between the flap pedicle and recipient vessels (Figure 2). Advantages of immediate reconstruction include fewer chest wall scars, preservation of the native breast skin envelope (Figure 1), a single hospitalization, and reduced psychological impact, as there is no interval during which the patient is without a breast.

Delayed reconstruction is typically undertaken after completion of oncologic treatment, including mastectomy, radiotherapy, and systemic therapy. Irradiated recipient vessels may be fragile and require meticulous dissection. Radiation also alters skin color and texture; consequently, the reconstructed breast may display color mismatch, with the flap skin paddle often appearing lighter and smoother than the surrounding irradiated skin. Scars are also generally longer and more conspicuous. A further option is immediate-delayed autologous reconstruction with a free flap. In this approach, a tissue expander is placed beneath the pectoralis major muscle at the time of mastectomy. During a second operation, the expander is removed, and definitive reconstruction with a free flap is performed after completion of oncologic therapy, including radiotherapy. The expander is gradually inflated in the outpatient setting between procedures to stretch the skin envelope, allowing use of a smaller skin paddle and resulting in shorter scars than in standard delayed reconstruction.

Free-flap reconstruction is typically performed by two reconstructive teams, which has reduced operative time from approximately six to eight hours to three to five hours. During immediate reconstruction, while the oncologic surgeon performs the mastectomy, one team prepares the donor tissue—most often from the abdomen—while the second team prepares the recipient vessels once the oncologic portion is completed. Microvascular anastomoses are then performed (Figure 2), followed by shaping and inset of the flap to create the breast mound.

## 10. Abdominal Free Flaps

**Figure 3:**
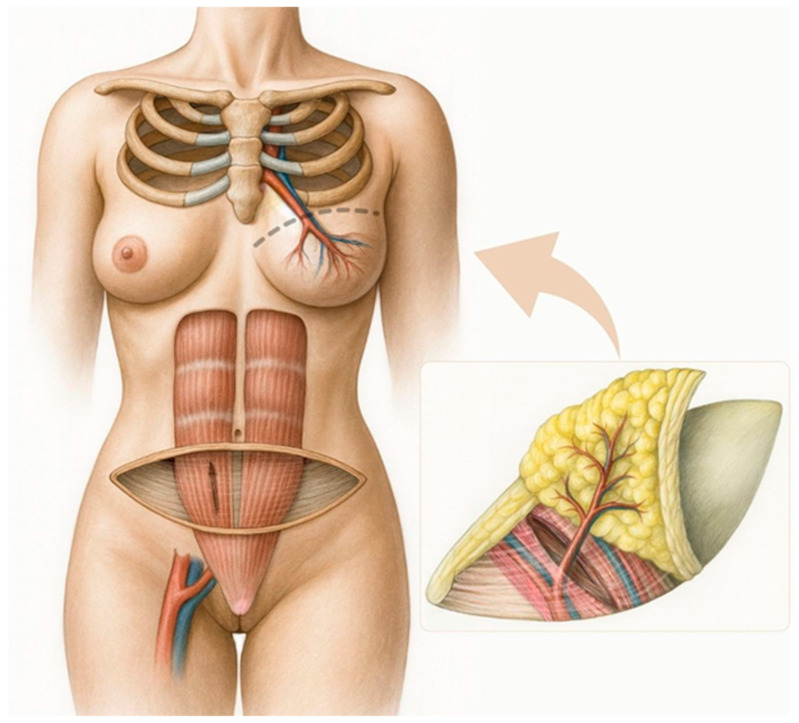
Autologous Breast Reconstruction with a Free Abdominal Flap (DIEP Flap). Note the perforators supplying the flap in the enlarged box on the right.

The arterial supply to the abdominal region used for flap harvest is provided by the paired deep inferior epigastric artery (DIEA) and the paired superficial inferior epigastric artery (SIEA). The DIEA courses beneath the rectus abdominis muscle, where its medial and lateral branches give rise to musculocutaneous perforators that traverse the muscle to supply the subcutaneous tissue and skin (Figure 3). In contrast, the SIEA runs within the subcutaneous plane. Both arterial systems are accompanied by comitant veins.

**Figure 4:**
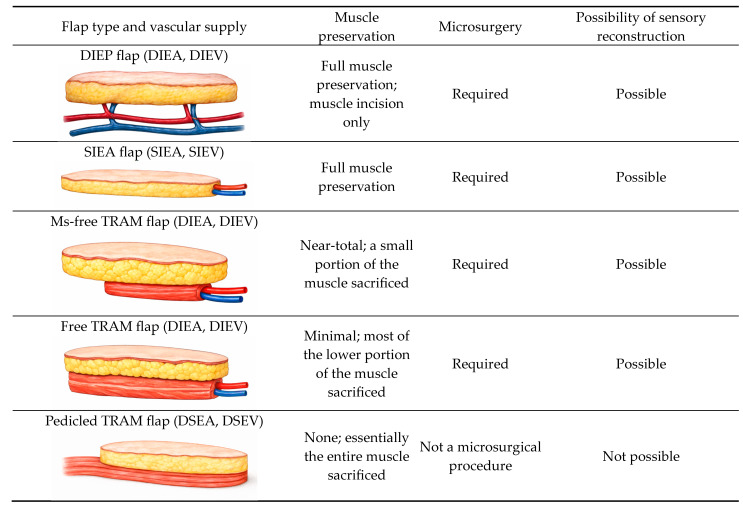
Comparison of different abdominal flaps.

In most cases, reconstruction is performed using a flap based on the deep system—the DIEP flap (Figure 3)—as the SIEA flap has several limitations. When an SIEA flap is used, the fascial envelope of the muscle does not need to be incised, eliminating the risk of postoperative hernia or abdominal wall weakness (Figure 4). However, the vascular pedicle obtained is typically short. Additional drawbacks include frequent anatomical absence of the SIEA, uncertainty regarding its ability to perfuse the contralateral hemiabdomen, and its smaller vessel caliber compared with the DIEA, which may complicate microvascular anastomosis between donor and recipient vessels.

A muscle-sparing abdominal flap may also be harvested (the traditional TRAM flap is now largely obsolete). The principal advantage of the DIEP flap lies in the preservation of the entire rectus muscle and its motor innervation (Figure 3, Figure 4). In rare cases, this is not feasible, and a small portion of muscle must be included to ensure adequate perfusion of the transferred tissue, in which case an ms-TRAM flap is performed (Figure 4).

At the University Medical Centre Ljubljana, the DIEP flap is practically always the planned abdominal free-flap option, owing to its lower donor-site morbidity and improved preservation of the rectus abdominis muscle (Figure 3, Figure 4). The ms-TRAM flap is not routinely preferred, but may occasionally serve as an intraoperative alternative when a planned DIEP flap cannot be safely completed. This is most likely in cases of unfavorable perforator anatomy, such as small, insufficient, or poorly distributed perforators, or when intramuscular pedicle dissection proves excessively complex and may compromise flap perfusion or surgical safety. In such situations, limited inclusion of muscle may provide a more reliable vascular supply while still aiming to minimize donor-site morbidity.

Contraindications to abdominal free-flap harvest are uncommon. Conditions such as smoking, arterial hypertension, and diabetes mellitus were previously considered contraindications, but this is no longer the case. Current contraindications are essentially limited to prior procedures that have compromised the abdominal vascular supply and insufficient donor-site tissue. Preoperative assessment routinely includes CT angiography to delineate the abdominal vasculature.

## 11. Complications of Free-Flap Breast Reconstruction

If a portion of the rectus abdominis muscle (together with its motor nerve supply) must be sacrificed during flap harvest, functional impairment may occur at the donor site. For this reason, a DIEP flap is preferred whenever feasible, and an ms-TRAM flap is used only when absolutely necessary [22,23]. In general, weakening of the abdominal wall is undesirable, as it may predispose to postoperative hernia formation.

At the recipient site, where microvascular anastomoses are performed, thrombosis is the principal concern, as it may result in flap loss. Should this occur, urgent re-exploration of the anastomosis patency is required. Postoperatively, ward staff therefore monitor flap color, capillary refill, and temperature at regular intervals. Additional modalities, such as handheld Doppler or implantable Doppler devices, may also be used to assess flap viability. Other possible complications include hypertrophic scarring, infection, seroma or hematoma formation, necrosis of the flap skin and/or adipose tissue, and umbilical necrosis [24]. Marginal skin necrosis of the flap (skin paddle) is observed occasionally, and areas of induration due to fat necrosis in relatively poorly perfused portions of the flap may also occur; however, these findings rarely necessitate additional intervention.

According to our clinical experience, partial flap loss (2–3%) or total flap loss (approximately 1%) is uncommon [25]. Necrotic tissue must be excised and the resulting defect reconstructed, most often using an alternative reconstructive method. In cases of vascular compromise at the microanastomotic site, revision microsurgery is required.

## 12. Autologous Breast Reconstruction with a Pedicled Flap

Pedicled flaps differ from free flaps in that their vascular supply remains intact throughout the transfer and is never divided. Owing to advances in microsurgical techniques in recent years, their use has declined substantially, and they are now rarely performed, particularly in our setting. Among pedicled flaps, the most commonly employed are the latissimus dorsi muscle or latissimus dorsi myocutaneous flap and the pedicled TRAM flap. The latissimus dorsi flap is used primarily for reconstruction of larger partial breast defects, but it may also be suitable for bilateral reconstruction in patients with small breasts and can be combined with an implant when additional volume is required. In implant-assisted reconstruction, it improves breast contour while providing well-vascularized soft-tissue coverage over the prosthesis. The pedicled TRAM flap is suitable even for reconstruction of large breasts. However, this technique may weaken the abdominal wall and is associated with an increased risk of postoperative hernia formation.

## 13. Breast Reconstruction Using Lipofilling

Breast reconstruction with lipofilling (autologous fat grafting) may be performed either as an adjunctive technique or, in selected cases, as a primary reconstructive modality [26]. The procedure involves harvesting adipose tissue via liposuction, processing it, and reinjecting it into the mastectomy site to gradually restore breast volume and contour [26]. When used as a standalone reconstruction, multiple staged sessions are typically required to achieve adequate volume [26]. Lipofilling offers the advantages of using autologous tissue, minimal donor-site morbidity, and improvement in skin quality, particularly in irradiated tissues; however, variable fat resorption and the need for repeated procedures remain its main limitations [26].

## 14. Reinnervation of the Breast Reconstructed with a Free Flap

Reconstruction with a free flap is widely considered the most effective method of breast reconstruction, as it provides the most natural aesthetic result and the greatest long-term durability. Functional outcomes may be further enhanced by performing neurorrhaphy between donor and recipient nerve ends. Autologous reconstruction using an innervated free flap has emerged over the past decade as an important refinement of microsurgical breast reconstruction [27,28,29,30,31]. In a systematic review, Beugels et al. [32] demonstrated significantly improved sensibility in reinnervated flaps compared with non-innervated reconstructions. Restoration of breast sensation has likewise been shown to have a meaningful positive impact on patient quality of life [33].

Sensory reinnervation of the reconstructed breast may improve quality of life not only through better sensory recovery itself, but also through measurable gains in psychosocial and sexual well-being, improved nipple sensation, and fewer postoperative breast symptoms. In a recent prospective cohort study [34], neurotized patients had significantly higher BREAST-Q psychosocial and sexual well-being scores, better nipple sensation, and fewer denervation-related symptoms, while improved objective sensory recovery was also significantly associated with better psychosocial well-being and higher subjective nipple and breast sensation.

During flap harvest, cutaneous branches of the intercostal nerves must be identified, as they typically accompany the perforating vessels. The deep inferior epigastric artery and vein are then dissected together with the selected cutaneous branch of the intercostal nerve. Recipient vessels are prepared by longitudinal splitting of the pectoralis major muscle and removal of a short segment of the cartilaginous portion of the third rib, which usually exposes the internal mammary artery and one or two accompanying veins. The anterior branch of the intercostal nerve is then identified and mobilized, where it pierces the pectoralis major in association with perforating branches of the internal mammary vessels. Microvascular anastomoses are performed first, followed by coaptation of the cutaneous nerve branch of the free flap to the anterior branch of the third intercostal nerve.

## 15. Implant-Based Breast Reconstruction

Breast reconstruction may also be performed using prosthetic materials. Implant-based reconstruction can be classified according to timing as immediate (primary) or delayed (secondary), and according to technique as either two-stage or single-stage reconstruction.

In clinical practice, the two-stage approach is more commonly employed. In the first stage, performed after mastectomy, a tissue expander is placed beneath the pectoralis major muscle. The expander is then gradually inflated with saline at regular outpatient visits, allowing progressive expansion of the overlying soft tissues. In the second stage, typically about six months later, the expander is exchanged for a permanent anatomically shaped silicone implant of the type routinely used in aesthetic breast augmentation.

If postoperative radiotherapy is anticipated, autologous reconstruction is generally recommended, as outcomes of implant-based reconstruction tend to be inferior in irradiated tissues. Radiation may lead to thinning and fibrosis of the skin and subcutaneous tissue, which adversely affects reconstructive results.

Implant-based breast reconstruction is recommended in the following situations: at the patient’s request, when autologous reconstruction is not feasible (e.g., insufficient abdominal donor tissue), when radiotherapy is not required, in patients with small breasts, when the patient desires a more youthful breast contour with simultaneous aesthetic adjustment of the contralateral breast, in prophylactic mastectomy performed in patients who are carriers of genetic mutations associated with a higher risk of developing breast cancer.

The principal advantages of this method compared with autologous reconstruction are the absence of additional donor-site scars, since no tissue harvest is required, as well as shorter operative time and a more rapid recovery.

## 16. Single-Stage Implant-Based Breast Reconstruction

Implant-based reconstruction can also be performed in a single stage. Interest in single-stage breast reconstruction has increased in recent years, largely owing to greater awareness of prophylactic mastectomy. An increasing number of patients are identified as carriers of BRCA1, BRCA2, and other pathogenic mutations associated with an elevated risk of breast cancer, and many of these patients elect to undergo risk-reducing mastectomy.

In 2006, Salzberg reintroduced the concept of immediate single-stage breast reconstruction [35]. Single-stage implant reconstruction represents an appropriate option for carefully selected patients, allowing definitive reconstruction to be completed in a single operation immediately following therapeutic or prophylactic mastectomy [35,36,37]. In selected cases, it has proven to be a safe and reliable alternative to traditional two-stage reconstruction [35,36,37,38].

Through a strategically placed incision, such as a lateral, inframammary, or periareolar incision, the oncologic surgeon accesses the breast tissue, performs the mastectomy, and preserves the nipple–areola complex. During the same procedure, the reconstructive surgeon places a silicone implant beneath the pectoralis major muscle. Creation of an appropriate implant pocket is essential. Laterally, dissection proceeds in the plane between the pectoralis major and minor muscles, extending inferiorly to the inframammary fold and medially toward the sternum to form the muscular pocket. Care must be taken to preserve perforating vessels encountered during dissection, as injury may compromise perfusion of the skin envelope.

**Figure 5:**
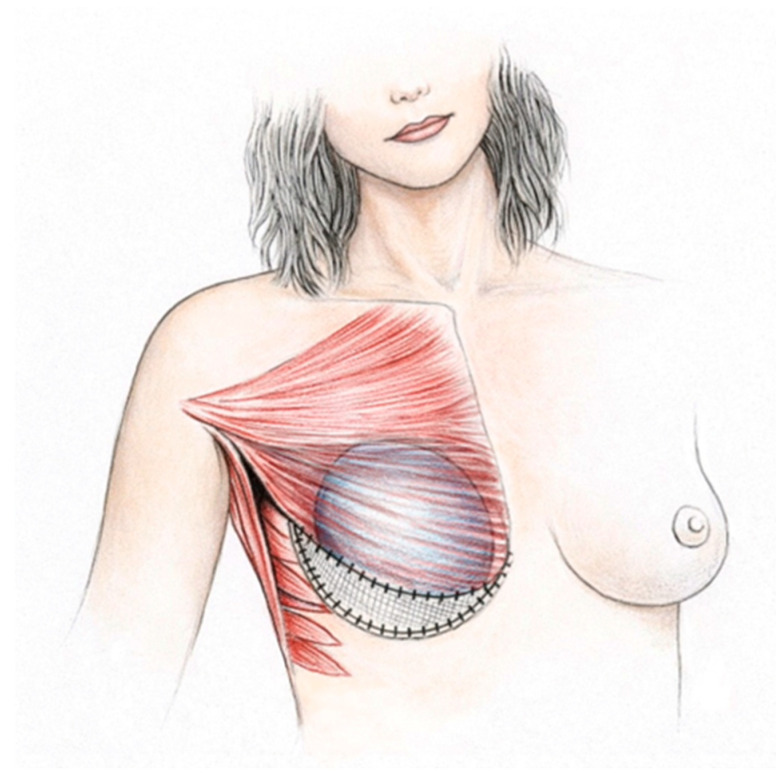
Direct-to-Implant Breast Reconstruction With Mesh.

Optimal implant positioning requires use of a supportive mesh placed in the lower pole of the breast, typically inferiorly and slightly laterally (Figure 5). A synthetic or biologic mesh is sutured to the inferior edge of the muscle to provide an additional soft-tissue layer (Figure 5). The implant is then inserted into the pocket, and the lower margin of the mesh is secured to the inframammary fold and lateral breast fold (Figure 5). In this configuration, the implant is covered partly by muscle and partly by mesh, which reduces palpability in the lower pole and helps maintain stable implant positioning (Figure 5).

Single-stage implant reconstruction is appropriate for carefully selected patients, particularly younger patients with BRCA1 or BRCA2 mutations or other genetic alterations conferring high breast cancer risk, and patients with early-stage disease in whom nipple–areola preservation is feasible at the time of mastectomy. Within this group, the approach is particularly suitable for patients who lack sufficient adipose tissue for autologous reconstruction, as well as for younger, lean, and physically active individuals who prefer a less invasive procedure and a shorter recovery period [36,39,40].

Single-stage reconstruction offers the advantage of immediate breast restoration at the time of mastectomy within a single operative session. Historically, the technique was largely abandoned because of complications such as muscle retraction, implant malposition, and capsular contracture. The introduction of acellular dermal matrices (ADMs), biologic meshes, provided a solution to many of these issues, and in 2006, Salzberg revived the concept of single-stage implant reconstruction using a biologic mesh [35]. ADM integrates into the surrounding tissue, thereby replacing deficient soft tissue. Composed of native extracellular matrix components, it is well tolerated and promotes neovascularization, leading to gradual remodeling into cellular tissue.

The mesh maintains tension of the pectoralis muscle and stabilizes the implant pocket, preserving its shape and volume while preventing implant displacement (Figure 5). Prior to 2006, biologic meshes had primarily been used for secondary correction following implant reconstruction to reduce skin rippling [41,42]. The first nonabsorbable synthetic mesh was introduced in 1997 [43], followed by the first absorbable synthetic mesh in 2014 [44].

Prepectoral implant-based reconstruction has gained increasing popularity in recent years. In this approach, the implant is placed above the pectoralis major muscle, within the prepectoral plane, thereby avoiding muscle dissection and reducing animation deformity and postoperative pain [45]. To provide implant support and improve soft tissue coverage, a biological mesh, most commonly an ADM, is used to cover the anterior surface of the implant, creating a stable and well-defined implant pocket [45].

Complications of single-stage implant reconstruction may include limited areas of skin necrosis, occasionally requiring minor surgical revision such as excision of necrotic tissue or lipofilling. Other possible complications include infection, capsular contracture, seroma or hematoma formation [46,47,48].

## 17. Two-Stage Implant-Based Breast Reconstruction

**Figure 6:**
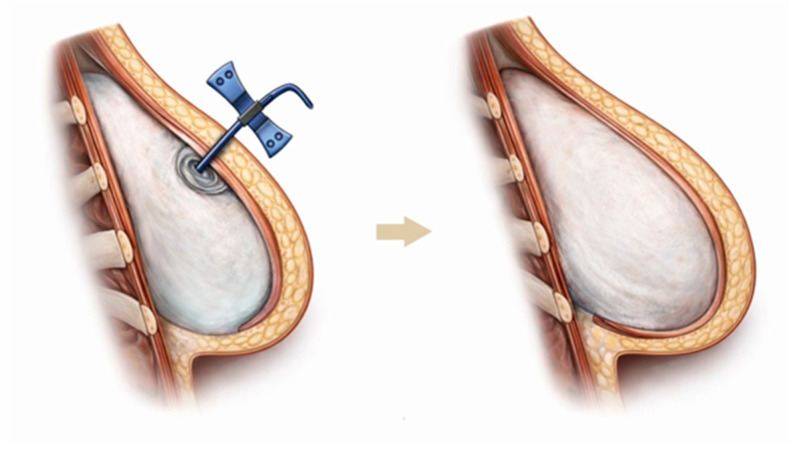
Two-Stage Implant-Based Breast Reconstruction.

Two-stage implant-based reconstruction begins with placement of a tissue expander, which is gradually inflated with saline during outpatient visits, allowing progressive expansion of the overlying tissues until sufficient volume is achieved for placement of a permanent anatomically shaped silicone implant of the type used in aesthetic breast augmentation (Figure 6). In most cases, a primary (immediate) two-stage reconstruction is performed, with the expander placed at the time of mastectomy.

After completion of the mastectomy, the reconstructive phase begins. The surgeon prepares a muscular pocket appropriate for the selected tissue expander. The lateral portion of the pectoralis major muscle is elevated, and dissection is continued medially toward its sternal insertion. The superior limit of the pocket lies at approximately the second to third rib, and the inferior limit corresponds to the inframammary fold. Because the pectoralis major alone cannot completely cover the expander, complete muscular coverage requires release of its costal attachments, elevation of the inferior slips of the serratus anterior muscle, and mobilization of the rectus sheath. Alternatively, coverage of the expander can be achieved using a synthetic or dermal mesh placed in the inferior and lateral regions. With this approach, muscular elevation is unnecessary, yet the expander remains covered in its lower and lateral aspects, resulting in improved aesthetic outcomes [49]. Without either muscular or mesh coverage, the expander would be partially covered only by skin and subcutaneous tissue, leading to inferior aesthetic results and increased palpability of the implant. The expander is partially filled at the time of insertion.

Expansion is typically initiated two to three weeks after surgery, with saline injections performed during outpatient visits. Usually, six to eight sessions are required to achieve adequate tissue expansion. The volume instilled at each visit depends on tissue compliance and the patient’s tolerance. Approximately six months after the final expansion, the expander is exchanged for a permanent silicone implant designed to resemble a natural breast. During this procedure, the fibrous capsule that has formed around the expander is partially or completely excised as needed to optimize the implant pocket.

Clinical experience in our practice indicates that the use of tissue expanders and implants is safe and associated with a low complication rate. Potential complications include implant rupture, seroma, hematoma, and capsular contracture [50,51,52,53,54]. Infection remains a frequent complication; skin necrosis and expander extrusion may also occur [50,51,52,53,54]. Radiotherapy administered as part of oncologic treatment increases the risk of suboptimal reconstructive outcomes [55]. Irradiation leads to thinning of the soft tissues, a change that is particularly evident in lean patients. Results may deteriorate over time, especially due to capsular contracture, which can distort implant shape and breast contour [55]. In such cases, revision surgery with capsular release is required.

Reported complication rates vary considerably across studies because they are influenced by differences in patient selection, mastectomy-flap perfusion, implant plane, the use of ADM or other coverage materials, and duration of follow-up. In a head-to-head meta-analysis, Basta et al. [53] reported pooled absolute complication rates for direct-to-implant versus conventional two-stage implant reconstruction of 7.8% versus 7.4% for infection, 6.8% versus 7.1% for seroma, 4.3% versus 5.2% for hematoma, 8.6% versus 6.7% for flap necrosis, 13.5% versus 13.8% for capsular contracture, 17.9% versus 14.1% for reoperation, and 14.4% versus 8.7% for implant loss. Notably, direct-to-implant reconstruction was associated with significantly higher risks of flap necrosis, reoperation, and implant loss, whereas infection, seroma, hematoma, and capsular contracture did not differ significantly between the two approaches. However, a more recent meta-analysis by van der Wielen et al. [54] did not identify significant differences between one-stage and two-stage implant-based reconstruction in overall complication rates.

## 18. Breast Implant-Associated Anaplastic Large Cell Lymphoma

In the context of breast reconstruction with implants, it is important to mention a rare form of T-cell lymphoma associated with breast implants, referred to as breast implant-associated anaplastic large cell lymphoma (BIA-ALCL). BIA-ALCL is a rare subtype of T-cell lymphoma that develops in association with textured breast implants. The disease typically presents 8–10 years after implant placement [56]. The pathogenesis of BIA-ALCL has not yet been fully elucidated; however, it is believed to be related to chronic inflammation with a sustained T-cell-mediated immune response [57].

According to estimates by Collett et al., the incidence of BIA-ALCL is approximately 1 in 2832 women with breast implants, while de Boer et al. report an incidence of 1 case per 6920 patients with implanted breast prostheses [58,59]. To date, all reported cases of this lymphoma have been associated with textured implants. In 2019, following recommendations by the FDA, Biocell textured breast implants—most frequently linked to the development of BIA-ALCL—were withdrawn from the market.

In the majority of cases, the initial clinical manifestation is a late seroma (a periprosthetic fluid collection) occurring more than one year after implant placement, unrelated to infection or implant rupture. This typically presents as breast swelling or unilateral breast enlargement. In a smaller proportion of patients, a palpable breast mass or regional lymphadenopathy may be observed. Cutaneous changes or capsular contracture occur only rarely.

Although breast implant-associated T-cell lymphoma is rare, patients should be informed about potential warning symptoms, as early recognition of BIA-ALCL is essential for successful treatment and favorable outcomes.

At the University Medical Centre Ljubljana, the 2019 withdrawal of Biocell implants did not lead to a reduction in implant-based breast reconstruction. In fact, these implants had already been abandoned in our institution before their formal withdrawal, although some other textured implants remain in use because their reported association with BIA-ALCL is considerably lower. Accordingly, the withdrawal had no meaningful effect on our reconstructive practice, and implant-based reconstruction remains an important option, particularly given that BIA-ALCL is a very rare complication associated predominantly with Biocell textured implants.

Beyond BIA-ALCL, concerns have also been raised regarding other potential systemic effects of breast implants. Breast implant illness (BII) is a term used to describe a range of nonspecific systemic symptoms attributed by some patients to their implants, including fatigue, arthralgia, myalgia, and cognitive impairment [60]. While a causal relationship has not been definitively established, symptom improvement has been reported following implant removal [60].

## 19. Nipple Reconstruction and Areolar Tattooing

Nipple reconstruction and areolar tattooing represent the final stage of breast reconstruction. In most patients, the nipple–areola complex is removed during skin-sparing mastectomy, as the nipple also contains glandular tissue and may therefore be involved in the disease process.

Nipple reconstruction is usually proposed at least six months after breast reconstruction, once all surgical wounds have healed and the reconstructed breast has achieved adequate ptosis and a stable shape. At the patient’s request, nipple reconstruction can be performed on an outpatient basis under local anesthesia using a local flap. The reconstructed nipple is intentionally created slightly larger than the desired final size, as some degree of flattening and volume loss is expected over time. One commonly used technique is the Thomas arrow flap [61], which is considered the most frequently applied method, as it is particularly effective in minimizing postoperative volume reduction and flattening of the reconstructed nipple. Nipple reconstruction is performed only after completion of oncological treatment and at least six months following breast reconstruction, allowing the reconstructed breast sufficient time to reach its final contour. Performing nipple reconstruction earlier may result in nipple malposition and asymmetry. In addition, oncological treatments may adversely affect the final aesthetic outcome of the reconstructed nipple [62].

To achieve the most accurate match in color and surface characteristics of the areola, areolar reconstruction is most commonly performed using medical tattooing, although skin grafts or a combination of both techniques may also be employed. Becker [63] was the first to propose tattooing of the reconstructed areola in 1986, while Spear [64,65] subsequently contributed to the wider adoption and recognition of this technique. Modern medical tattooing devices are of high quality and offer a wide range of pigments and shades, enabling optimal color matching with the contralateral, healthy areola. Areolar tattooing is typically performed 6–8 weeks after nipple reconstruction and is carried out by a specially trained nurse. In some cases, additional tattooing may be required, as pigment fading can occur over time. Given that tattoo pigmentation tends to fade gradually, the use of a higher pigment concentration during the initial procedure is recommended [65].

It should be noted that sensation in the reconstructed nipple–areola complex is reduced, and the reconstructed nipple lacks erectile function.

## 20. Emerging and Evolving Techniques in Breast Reconstruction

Robotic techniques have gradually expanded into both reconstructive and ablative breast surgery, particularly in robot-assisted autologous flap harvest and robotic nipple-sparing mastectomy (RNSM). Available evidence suggests that robotic autologous breast reconstruction, most commonly involving DIEP and latissimus dorsi flap harvest, is technically feasible and associated with postoperative complication rates comparable to those of conventional surgery, although usually at the cost of longer operative times [66,67,68,69]. At the same time, some systematic reviews and meta-analyses have reported a shorter hospital stay in selected cohorts, supporting the potential of robotic reconstruction as a minimally invasive adjunct in carefully selected patients [66,67,68,69]. A similar pattern has been observed for RNSM, where the available literature indicates acceptable short-term surgical and oncologic outcomes, with immediate reconstruction performed in most reported cases [70]. More recent meta-analytic evidence further suggests that RNSM may reduce nipple necrosis compared with conventional nipple-sparing mastectomy, although this possible advantage should be interpreted cautiously, given the longer operative duration and continued lack of robust long-term outcome data [71].

Hybrid breast reconstruction has emerged as an increasingly valuable strategy for patients in whom implant-based reconstruction alone may provide insufficient soft-tissue coverage or suboptimal contour, particularly in the setting of thin mastectomy flaps, prior radiotherapy, or a marked mismatch between desired breast volume and available donor tissue [72,73]. This approach most commonly combines prosthetic reconstruction with autologous tissue augmentation—either through fat grafting or flap-based techniques—to distribute volume in a planned manner and improve both contour and soft-tissue coverage [72,73]. Available evidence suggests that hybrid reconstruction may improve implant camouflage, reduce visible rippling, and enhance overall aesthetic outcomes while maintaining an acceptable complication profile, although the current literature remains heterogeneous and is still dominated by retrospective series rather than high-level comparative trials [72,73,74]. Current evidence has also emphasized the expanding role of prepectoral implant placement, individualized volume sharing between prosthetic and autologous components, and the particular utility of hybrid reconstruction in patients with limited donor tissue or anticipated radiotherapy, thereby supporting its growing role within contemporary breast reconstruction practice [72,73,74]. 

Fat-augmented latissimus dorsi (FALD) flap reconstruction has re-emerged as a valuable autologous option for selected patients who are poor candidates for abdominal free flaps, wish to avoid abdominal donor-site morbidity, or require an alternative strategy after previous irradiation [75,76,77]. The available evidence suggests that FALD-based reconstruction is technically feasible and capable of achieving satisfactory breast volume and contour through immediate fat grafting to the latissimus dorsi flap, thereby reducing or avoiding the need for an implant in appropriately selected patients [75,76]. A systematic review and meta-analysis by Escandón et al. [75] supported the safety and reconstructive utility of this approach, although immediate fat transfer did not eliminate the frequent need for subsequent lipofilling; a more recent systematic review by Tilkin et al. [76] further suggested that FALD is a safe alternative to implant-assisted latissimus dorsi reconstruction, with a possible trend toward lower major complication rates.

## 21. Comparative Considerations Across Reconstructive Techniques

**Autologous versus Implant-Based Reconstruction**

Current comparative evidence suggests that autologous breast reconstruction, most commonly performed today using free-flap techniques such as the DIEP flap, generally achieves superior long-term patient-reported outcomes and higher satisfaction with the reconstructed breast compared with implant-based reconstruction. This advantage is attributed to the use of vascularized autologous tissue, which provides a more natural breast contour and long-term adaptability to body changes, meaning that the reconstructed breast tends to age and change more naturally with the body over time, including with weight fluctuation, while avoiding long-term implant-related problems such as rupture, malposition, infection, rippling, or capsular contracture. For many patients, abdominal flap harvest may offer an additional aesthetic benefit by simultaneously improving abdominal wall contour, an effect comparable to that achieved with abdominoplasty in aesthetic surgery.

At the same time, these benefits must be balanced against the greater complexity of microsurgical flap transfer, longer operative times, longer recovery, and the risk of donor-site morbidity, including the risk of abdominal wall weakness, bulge, or hernia in abdominally based flap reconstruction. Thus, autologous reconstruction may offer superior long-term aesthetic and patient-reported outcomes, but at the cost of greater procedural burden and potential functional sequelae related to flap harvest. Meta-analytic evidence supports this trade-off: in a systematic review and meta-analysis of BREAST-Q patient-reported outcomes, Toyserkani et al. [78]. compared autologous and implant-based breast reconstruction across five commonly reported domains—satisfaction with the breast, satisfaction with outcome, psychosocial well-being, sexual well-being, and physical well-being—and found higher satisfaction with the breast and overall outcome after autologous reconstruction, together with better psychosocial and sexual well-being, whereas physical well-being, which reflects patient-reported chest and upper-body pain, tightness, discomfort, and activity-related physical limitations, was comparable between the two approaches. Stefura et al. [79], in a systematic review and meta-analysis, similarly reported higher BREAST-Q–based satisfaction with breast aesthetics and with the overall reconstructive treatment after autologous reconstruction, while safety outcomes were broadly comparable and treatment costs were higher.

In addition, autologous reconstruction is generally preferred in patients expected to receive radiotherapy or in those with a previously irradiated breast, because it tends to provide more stable long-term outcomes in compromised tissues [80,81,82].

By contrast, implant-based reconstruction is generally less invasive, requires shorter operative time, allows faster recovery, and avoids donor-site sacrifice, which explains why it remains the most commonly performed reconstructive approach in many settings; however, it is more dependent on implant-related long-term outcomes and may require revisional surgery over time.

**Single-Stage versus Two-Stage Implant-Based Reconstruction**

Within implant-based reconstruction itself, however, current evidence does not demonstrate a clear overall superiority of one-stage direct-to-implant reconstruction over the traditional two-stage expander-to-implant approach. Rather, recent meta-analytic data suggest similar patient satisfaction, aesthetic outcomes, and complication rates, suggesting that one-stage reconstruction offers the main advantage of avoiding a second operation and is generally associated with lower overall treatment costs when ADM, which substantially increases procedural cost, is not used. However, this approach requires careful patient selection, adequate mastectomy flap perfusion, and a well-preserved skin envelope, ideally with preservation of the nipple–areola complex. The two-stage reconstruction may still be preferable when skin envelope quality, mastectomy flap perfusion, or oncologic circumstances make a staged approach safer and more predictable [54].

Taken together, when the skin envelope is preserved, ideally together with the nipple–areola complex, and mastectomy flap perfusion is adequate, comparable patient satisfaction, aesthetic outcomes, and complication rates support the use of one-stage breast reconstruction in these carefully selected patients [54].

With regard to the overall duration of treatment and patient experience, two-stage implant-based reconstruction is generally more prolonged and burdensome, as it requires not only two separate operations but also multiple outpatient visits between procedures for gradual tissue expansion. During this phase, the expander is progressively filled with saline, which stretches the overlying tissues and may cause chest tightness, discomfort, and pain.

Overall, the literature supports autologous reconstruction as the option most consistently associated with higher long-term satisfaction, whereas implant-based strategies remain highly valuable because of their lower procedural burden. Within implant-based reconstruction, the choice between one-stage and two-stage approaches should be individualized according to patient characteristics, tissue conditions, and adjuvant treatment plans.

**Prepectoral versus Subpectoral Implant Placement**

When comparing prepectoral with subpectoral implant placement, the main trade-off is between reduced muscle-related morbidity and the need for adequate soft-tissue coverage. Because the prepectoral approach preserves the pectoralis major muscle, it has been associated with less postoperative pain, less functional impairment, and avoidance of animation deformity, making it an increasingly attractive option in appropriately selected patients [83,84].

At the same time, comparative studies suggest that overall complication profiles are broadly similar between the two planes, although specific complications may differ. In a recent systematic review and meta-analysis, Ostapenko et al. [83] reported significantly lower rates of capsular contracture, prosthesis failure, and animation deformity with prepectoral reconstruction, whereas other complications and overall BREAST-Q scores did not differ significantly between the two approaches. Another systematic review and meta-analysis by Mégevand et al. [84]. likewise found comparable overall postoperative complications, while noting higher pain scores after subpectoral placement and a higher rate of seroma after prepectoral reconstruction.

In a recent meta-analysis, Wu et al. [85] found that prepectoral and subpectoral implant-based reconstruction were associated with similar overall complication rates, with no significant differences in most individual postoperative complications. Nevertheless, prepectoral reconstruction showed a distinct complication profile, with significantly higher rates of seroma and rippling, but a lower rate of animation deformity compared with subpectoral placement. These findings indicate that, although the two approaches appear broadly comparable in terms of overall safety, they differ in the frequency of specific complications, which should be taken into account during patient selection and preoperative counseling.

Nolan et al. [86], in a systematic review and meta-analysis focused on immediate implant-based reconstruction after nipple-sparing mastectomy, found that prepectoral reconstruction was associated with a lower rate of mastectomy-flap necrosis, while other consistently reported complications, including hematoma, seroma, infection, nipple–areola complex necrosis, and explantation, were broadly comparable between the two approaches. However, the lower flap-necrosis rate should be interpreted cautiously, as the authors noted that this finding may have been influenced by selection bias, given that prepectoral reconstruction is typically reserved for patients with adequately perfused mastectomy flaps. In the broader descriptive analysis, rippling was reported more frequently after prepectoral reconstruction, although it was not among the consistently reported outcomes included in the formal comparative meta-analysis. Overall, these findings support the safety of immediate prepectoral reconstruction in appropriately selected patients.

Similarly, Pumilia et al. [87], in a more recent systematic review and meta-analysis, reported broadly comparable overall complication profiles between prepectoral and submuscular reconstruction. However, the distribution of specific complications differed: prepectoral reconstruction was associated with higher rates of seroma and rippling, whereas animation deformity was more frequent after submuscular placement. This further suggests that the choice of implant plane should be individualized according to soft-tissue conditions and the balance between a lower risk of animation deformity and a higher risk of seroma and rippling.

To conclude, recent meta-analytic data support a lower risk of animation deformity with prepectoral reconstruction, but suggest higher rates of rippling and seroma, highlighting the importance of careful patient selection and adequate mastectomy-flap quality when this approach is chosen. Accordingly, prepectoral reconstruction appears particularly advantageous in well-selected patients with sufficiently thick, well-perfused mastectomy flaps, whereas subpectoral placement may still be preferable when additional soft-tissue coverage is required.

**Considerations for the Use of ADM in Prepectoral Reconstruction**

Across the prepectoral-versus-subpectoral meta-analyses discussed above, ADM was part of the reconstructive context to varying degrees, but it was not consistently isolated as a separate variable; therefore, those plane-comparison analyses cannot by themselves determine the independent effect of ADM use.

Whether ADM should be used routinely in prepectoral breast reconstruction remains unsettled. Although ADM has played an important role in the adoption of the prepectoral approach by helping define the implant pocket and provide additional soft-tissue support, current comparative meta-analytic evidence does not demonstrate a clear universal reduction in complications when ADM is added. In the systematic review and meta-analysis by Nolan et al. [88] no significant differences were found between ADM-assisted and ADM-free prepectoral reconstruction in overall complication rates or in seroma, infection, capsular contracture, and rippling. The authors noted, however, that the no-ADM cohorts largely reflected carefully selected patients with robust, well-perfused mastectomy flaps.

Carrillo-Gamboa et al. [89], in a recent systematic review and meta-analysis, found broadly similar postoperative outcomes between the two approaches. In particular, they reported no significant differences in assessed complications, including reoperation, infection, seroma, hematoma, necrosis, implant removal, rippling, and capsular contracture. These findings suggest that ADM is not mandatory in all prepectoral reconstructions and that satisfactory outcomes may also be achieved without it in appropriately selected patients. At the same time, the authors note that ADM may still provide structural and aesthetic benefits in selected cases, supporting a selective rather than routine approach to its use.

Taken together, these findings suggest that ADM is not mandatory in all prepectoral reconstructions, but is best used selectively, with the decision guided by mastectomy-flap viability and thickness, the need for additional implant support, and cost considerations.

## 22. Psychological and Quality-of-Life Impact of Breast Reconstruction

Breast reconstruction following mastectomy has important implications not only for physical restoration but also for psychological well-being, body image, and overall quality of life. Chen et al. [90], in a 2018 meta-analysis, found that breast reconstruction after mastectomy was associated with significantly lower rates and severity of anxiety and depression than mastectomy alone. Pačarić et al. [91], in a prospective study, likewise reported poorer quality of life in patients who did not undergo reconstruction compared with those who received either immediate or delayed breast reconstruction, although no significant overall difference in quality of life was observed between the immediate and delayed reconstruction groups. These findings are broadly consistent with the large 2024 systematic review by Roy et al. [92], in which most included studies associated breast reconstruction after mastectomy with better psychological outcomes than mastectomy alone, while also suggesting a possible psychological advantage of immediate over delayed reconstruction.

In addition, comparative patient-reported outcome data indicate that psychosocial well-being, sexual well-being, and satisfaction with the reconstructed breast may vary according to reconstructive modality, with some studies showing higher satisfaction with autologous reconstruction than with implant-based techniques [78]. Additionally, postoperative quality of life is influenced not only by the reconstructive procedure itself, but also by complications, adjuvant treatment, preoperative expectations, and psychosocial factors, highlighting the importance of individualized decision-making and realistic preoperative counseling [93].

## 23. Conclusions

The evolution of mastectomy techniques has been closely accompanied by advances in breast reconstruction. As a result, breast reconstruction has become the final step in the comprehensive treatment pathway for breast cancer following mastectomy. At the University Medical Centre Ljubljana, Slovenia, longstanding collaboration between plastic surgeons and oncologists ensures access to high-quality reconstructive options that align with international standards.

An optimal breast reconstruction contributes substantially to a patient’s rehabilitation—not only in terms of physical restoration but, even more importantly, in supporting psychological recovery, rebuilding self-image, and reinforcing self-worth. Thus, full rehabilitation after breast cancer extends beyond oncological and surgical management alone; integrating psychological support into the treatment process would likely bring significant additional benefit. This represents an important challenge and opportunity for the future of modern breast cancer care.

## Data Availability

No new data were created or analyzed in this study.
